# Understanding the Effects of Adding Metal Oxides to Polylactic Acid and Polylactic Acid Blends on Mechanical and Rheological Behaviour, Wettability, and Photo-Oxidation Resistance

**DOI:** 10.3390/polym16070922

**Published:** 2024-03-27

**Authors:** Elisabetta Morici, Giuseppe Pecoraro, Sabrina Carola Carroccio, Elena Bruno, Paola Scarfato, Giovanni Filippone, Nadka Tz. Dintcheva

**Affiliations:** 1 ATEN Center, Università di Palermo, Viale delle Scienze, Ed. 18, 90128 Palermo, Italy; 2 Dipartimento di Ingegneria, Università di Palermo, Viale delle Scienze, Ed. 6, 90128 Palermo, Italy; 3 CNR-IPCB, Unit of Catania, Via P. Gaifami 18, 95126 Catania, Italy; sabrinacarola.carroccio@cnr.it; 4 CNR-IMM, Via Santa Sofia 64, 95123 Catania, Italy; elena.bruno@dfa.unict.it; 5 Dipartimento di Fisica e Astronomia “Ettore Majorana”, Università di Catania, 95124 Catania, Italy; 6 Department of Industrial Engineering, University of Salerno, Via Giovanni Paolo II, 84084 Fisciano (SA), Italy; pscarfato@unisa.it; 7 Dipartimento di Ingegneria Chimica, dei Materiali e della Produzione Industriale, Università degli Studi di Napoli Federico II, 80125 Naples, Italy; gfilippo@unina.it

**Keywords:** biopolymers, biopolymer blends, metal oxides, morphology refinement, photo-sensitive degradant agents

## Abstract

Biopolymers are of growing interest, but to improve some of their poor properties and performance, the formulation of bio-based blends and/or adding of nanoparticles is required. For this purpose, in this work, two different metal oxides, namely zinc oxide (ZnO) and titanium dioxide (TiO_2_), at different concentrations (0.5, 1, and 2%wt.) were added in polylactic acid (PLA) and polylactic acid/polyamide 11 (PLA/PA11) blends to establish their effects on solid-state properties, morphology, melt behaviour, and photo-oxidation resistance. It seems that the addition of ZnO in PLA leads to a significant reduction in its rigidity, probably due to an inefficient dispersion in the melt state, while the addition of TiO_2_ does not penalize PLA rigidity. Interestingly, the addition of both ZnO and TiO_2_ in the PLA/PA11 blend has a positive effect on the rigidity because of blend morphology refinement and leads to a slight increase in film hydrophobicity. The photo-oxidation resistance of the neat PLA and PLA/PA11 blend is significantly reduced due to the presence of both metal oxides, and this must be considered when designing potential applications. The last results suggest that both metal oxides could be considered photo-sensitive degradant agents for biopolymer and biopolymer blends.

## 1. Introduction

Metal oxide-based polymeric nanocomposites are a significant class of materials from a technological and scientific view. Incorporating metal oxides into the polymer matrix could improve physicochemical properties such as mechanical, thermal, biological, electrical, optical, and superficial properties [1,2]. The resultant nanocomposites are principally employed as sensors for the photodegradation of pollutants, in water treatment, in membrane and ion exchanger technologies, and as an adsorbent medium [3,4,5,6]. Among the different metal oxides, zinc oxide (ZnO) and titanium dioxide (TiO_2_) are largely used as fillers for polymer nanocomposite preparation [7,8]. Zinc oxide is a chemically accessible, low-cost, with a high redox potential, able to remove organic pollutants and acts as an anti-bacterial agent [9,10].

Titanium dioxide (TiO_2_) has been extensively researched due to its stable structure, non-toxicity, anti-corrosion properties, and high photocatalytic activity. Indeed, TiO_2_, both with ZnO nanoparticles, shows photocatalytic activity because of a positive band position that generates holes (h+) and electrons (e−) by molecular excitation under UV light, resulting then in the generation of hydroxyl radicals and reactive oxygen species which, in turn, can interfere with pollutants and bacterial metabolism inhibiting their growth or causing the bacterial death [11,12,13].

In addition to zinc oxide (ZnO) and titanium dioxide (TiO_2_), nanoparticles of copper oxide (CuO), tin dioxide (SnO_2_), and silver oxide (Ag_2_O) are currently being proposed for potential use in food packaging applications. These metal oxides have been shown to modify the optical, structural, electrical, and antibacterial properties of different polymer and biopolymer host matrices, making them much more suitable for advanced packaging applications [14,15,16,17].

The research community has been focused on the petroleum-derived polymer matrix. However, this traditional approach is not more sustainable because of the increase in plastic waste generation and accumulation and the growing alarms about the environmental impact of chemicals from petrochemical resources. These nanocomposites are non-biodegradable and can take many years to decompose, leading to worrying pollution and concern for human health and ecosystem stability [18]. The breakdown of larger plastics released into the environment produces microplastics (MPs), i.e., plastic particles less than five millimetres long, that are harmful to living organisms [18,19]. In addition, the manufacture of petrochemicals affects the environment by adding contaminants to the air, water, and soil and emitting greenhouse gases that can contribute to global climate change [20,21]. These troubles, combined with the shortage of oil resources, have motivated research in utilizing alternative sources of raw materials to produce a new class of polymers with a natural origin and degradability. In particular, biopolymers are supposed to replace the conventional polymeric matrix with the goal of zero-waste, lower energy requirement to manufacture, and zero-emission and no hazardous chemicals released during manufacturing processes. Moreover, biodegradable polymers are suitable for some biological and medical applications [22,23,24].

Poly(lactic acid) (PLA), a linear aliphatic compostable polyester, is synthesized from renewable raw materials and finds several applications in different fields due to its relatively low cost, safety, versatility, appealing mechanical properties, high clarity, and ease of processing. Furthermore, products from PLA degradation are safe for living entities and do not harm the environment. Its main uses are in food packaging, medical, automotive, textile, and agriculture areas [25,26,27]. Moreover, the production of polylactic acid, according to the production technique by the company NatureWorks, is expected to result in a 68% reduction in the consumption of fossil fuels compared to conventional plastics [28]. Despite these advantages, some properties need to be improved to achieve widespread use in industrial manufacturing. Critical drawbacks are related to brittleness, proper barrier properties, low melt strength, and thermal stability, while the degradation rate is slower compared to other common natural organic wastes, such as food and yard waste. The addition of nanoparticles (NPs) proved to be a method to overcome some limitations: significant improvements were observed in hybrid PLA nanocomposites, even at very low nanofiller loadings [29,30,31]. Physical thermomechanical properties enhance above all when proper polymer–filler interactions or suitable filler distribution are achieved.

Another successful, solvent-free, and cost-effective way to obtain improved properties of the material is by blending polymers with complementary properties. Polyamide 11 (PA11) is a bio-based polymer with a similar glass transition temperature and melting point to PLA that exhibits chemical resistance, high ductility and impact strength, and better thermomechanical properties compared to PLA, which makes it a suitable candidate for blending with PLA [32,33]. Although the energy demand for the production of PA11 is relatively high, it is lower than that of polyamide 66 (PA66) and other oil-based competitors in the same performance range, such as Polycarbonate (PC), polyamide 6 (PA6) or Poly(methyl methacrylate) (PMMA) [34]. Adding nanoparticles to immiscible blends could improve even more properties.

Although the presence of nanometric particles in polymers and biopolymers could be considered potentially harmful if the particles are well incorporated into the polymer or biopolymer matrices, their migration is not favoured, and the nanoparticles can explain their beneficial effects on the composite performance. Therefore, there could be a potential negative effect on landfill disposal at the end-of-life of the micro-/nano-composites, and this highlights the need for accurate separation and management of these materials. The micro-/nano-composite materials cannot be mixed with poor polymer or biopolymer waste and must be separated for efficient and safe recycling [35].

In this work, we present data that can be useful for materials science, aiming to find solutions to overcome drawbacks that limit applications of biopolymer-based nanocomposites. We study different PLA-based nanocomposites obtained, varying the metal oxide concentration as well as the type of matrix, i.e., pure PLA and the PLA/PA11 blend. At first, PLA/PA11 blends were prepared by mixing different amounts of the two homopolymers. Finally, the fully bio-based blend (PLA 70%wt. and PA11 30%wt.) containing the investigated metal oxide (ZnO and TiO_2_ at 0.5%wt., respectively) was studied.

## 2. Materials and Methods

### 2.1. Materials

The PolyLactic Acid (PLA) used in this work was a commercial extrusion sheet grade supplied by NatureWorks (Blair, NE, USA; named PLA 2002D) with an average molecular weight number of about 121,000 g/mol, a ratio of 96% L-lactide to 4% D-lactide units, and a melt flow index of 6 g/10 min (230 °C, 2.16 kg).

The polyamide used in this work was a Polyamide 11, PA11 (Nylon 11, pellets form, from Sigma Aldrich, St. Louis, MO, USA), with glass transition temperature Tg = 46 °C, melting temperature Tm = 198 °C, and density ρ = 1.026 g/cm^3^ at 25 °C; molecular weight: Mw = 201.31 g/mol, MFI@235 °C/2.16 kg = 14.5 ± 1.2 g/10 min.

Zinc oxide (ZnO) nanopowder (<100 nm particle size) was purchased from Sigma-Aldrich and used with further purification. According to our previous investigations, ZnO nanoparticle size spanned a 100–200 nm range, with a mix of tubular and round-shaped forms [35].

Titanium dioxide (TiO_2_, AEROXIDE^®^ TiO_2_ P25) was purchased from Evonic (Essen, Germany) and used with further purification. Specific surface area (BET): 35–65 m^2^/g; density: approx. 140 g/L.

### 2.2. Melt Processing

Both biopolymers PLA and PA11 and metal oxides were vacuum-dried at 80 °C overnight before processing to avoid hydrolytic degradation during melt processing. Neat PLA, containing commercial metal oxides (ZnO and TiO_2_) at varying amounts (0.5, 1, and 2%wt.) and PLA/PA11 blends, containing 0.5%wt. of metal oxides were prepared by melt mixing using a Brabender PLA-330 internal mixer at 200 °C for 5 min at 50 rpm. To improve the ZnO and TiO_2_ dispersion in the PLA melt and PLA/PA11 blend melt, the samples were pre-mixed for 1 min, and then ZnO was added, and the mixing continued for another 4 min.

Thin films, with a thickness of ca. 120 microns, which were requested for the characterizations were obtained by the hot compression moulding process.

### 2.3. Characterizations

#### 2.3.1. FTIR Spectroscopy

A Fourier Transform Infrared Spectrometer (Spectrum One, Perkin Elmer, Shelton, CT, USA) was used to record IR spectra using 16 scans at a resolution of 1 cm^−1^. The progress of photo-oxidation degradation of the samples has been followed by FTIR analysis monitoring the variations in carbonyl range (1850–1600 cm^−1^) in time, using Spectrum One (vers. 10.4.2.) software.

#### 2.3.2. Water Contact Angle (WCA)

The water contact angle (WCA) was measured at room temperature using the First Ten Angstrom (USA) FTA1000C system (Data Physics Instruments, Filderstadt, Germany) with demineralized water. The films were fixed on top of a plane solid support and kept flat during water deposition and acquisition. The sessile drop method was used with a droplet volume of 6 μL.

#### 2.3.3. Tensile Tests

Tensile tests were carried out using a universal testing machine (Instron model 3365, Bucks, UK), according to the ASTM D882 method, on rectangular samples. The tests were performed using a tensile speed of 1 mm/min for 1 min to evaluate Young’s modulus, and then the velocity was increased to 10 mm/min until sample breakage.

#### 2.3.4. Rheological Analysis

Rheological tests were performed using a stress-controlled rheometer (ARES G-2) in parallel plate geometry (plate diameter 25 mm). The complex viscosity (η*), storage (G′), and loss (G″) moduli were measured under frequency scans from ω = 10^−1^ to 100 rad/s at T = 170 °C. The strain amplitude was γ = 5%, which preliminary strain sweep experiments proved to be low enough to be in the linear viscoelastic regime.

#### 2.3.5. Scanning Electron Microscopy (SEM)

The morphologies of the blend nanocomposites were investigated by scanning electron microscopy images (SEM, Gemini 152 field emission SEM Supra 25, Carl Zeiss, Oberkochen, Germany). The images were obtained via the Inlens mode at 5 kV. The samples of neat PLA and PLA/PA11 were cryogenically fractured in liquid nitrogen to obtain a cross-section and subsequently sputter-coated with gold (10 mA, 4 min) to create a conductive surface layer of 10 nm.

The average numerical diameter (*d_n_*), Equation (1), average volumetric diameter (*d_v_*), Equation (2), and dispersion (*D*), Equation (3), of the diameters of the dispersed phase, were determined by measurements on the obtained SEM images (considering at least one hundred particles for each sample), containing more than one hundred (Σ*_i_*) dispersed phase particles per sample, according to the following equations:

(1) $$d_{n}=\frac{\Sigma_{i}n_{i}d_{i}}{\Sigma_{i}n_{i}}$$

(2) $$d_{v}=\frac{\Sigma_{i}n_{i}d_{i}^{4}}{\Sigma_{i}n_{i}d_{i}^{3}}$$

(3) $$D=\frac{d_{v}}{d_{n}}$$

For particles with an ellipse form, an equivalent diameter, Equation (4), was used via the particle area, according to the following equation:

(4) $$d_{eq}=2{\left(\frac{A}{\pi }\right)}^{\frac{1}{2}}$$

#### 2.3.6. Differential Scanning Calorimetry (DSC)

Calorimetric analysis was evaluated by differential scanning calorimetry (DSC), using a TA Instruments Q100 in a nitrogen atmosphere on 5 ± 0.5 mg samples sealed in aluminum crucibles. Samples were heated from ambient temperature to 230 °C at 10 °C/min, and held in equilibrium conditions for 3 min at 230 °C to eliminate the thermal history, cooled to 25 °C at 10 °C/min, and then reheated to 230 °C at 10 °C/min.

### 2.4. Photo-Oxidation Exposure

Accelerated photooxidation was carried out using a Q-UV/basic weatherometer (from Q-LAB, Westlake, OH, USA) equipped with UVB lamps (313 nm). The weathering conditions consist of a continuous light irradiation at T = 70 °C.

## 3. Results and Discussion

### 3.1. Effect of ZnO and TiO_2_ Adding on PLA Properties

As a preliminary study, two different metal oxides, such as zinc oxide (ZnO) and titanium dioxide (TiO_2_), at different concentrations, i.e., 0.5, 1, and 2%wt, were added in PLA to establish their effect on solid state properties, melt behaviour, and photo-oxidation resistance. 

In Table 1, the mechanical properties of PLA and PLA-based systems, i.e., tensile or Young’s modulus (E) tensile strength (TS), and elongation at break (EB), at different oxide loadings are reported. The Young’s modulus of the PLA/ZnO bio-composite decreased with respect to the pure matrix and as the ZnO contents increased. The results show that adding TiO_2_ decreases Young’s modulus of PLA from 980 MPa to about 745 MPa, and the values exhibit quite different values as the oxide content increases. The effect of both oxides on the tensile strength of the neat matrix is almost the same: at lower oxide concentrations, no relevant change in value was recorded; at high concentrations, the values decrease with the same trend for both oxides. Otherwise, the elongation at the break of PLA increases in the presence of TiO_2_ while decreasing in the presence of ZnO.

Proper dispersion of the nanoparticles into the polymer matrix ensures an effective stress transfer from the matrix to the filler and is crucial to obtain a reinforcing effect, while a poor dispersion and the formation of aggregate acting as stress concentrators lead to the weakening of the mechanical stability [36]. The incompatible nature between the mineral and organic phases and the inherent tendency of oxide nanoparticles to aggregate due to van der Waals interactions and high surface free energy related to the metallic bonds could cause defects in the matrix, reducing the ultimate tensile strength., so the incorporation of ZnO and TiO_2_ into a PLA matrix could cause a decrease in the values of TS. Moreover, the ZnO oxide could accelerate the degradation mechanism of PLA during preparation, as confirmed by further analysis, leading to lower molecular weight of the matrix and worsening the mechanical properties. The effect of the addition of TiO_2_ on the thermal stability of PLA during preparation seems to be not impactful; additionally, TiO_2_ seems to improve the mobility of matrix chains in the solid state and shows a slight lubricant effect for the PLA matrix.

Table 1 also reported the water contact angle measurements: the presence of metal oxide can modify the surface properties, including the hydrophobicity, an important feature in packaging applications since the value of WCA is correlated to the resistance to humidity. The water contact angle measured for neat PLA is 60.1°. The addition of both investigated oxides causes an increase in water contact angle. The TiO_2_ presence at 2 wt% makes the PLA surface more hydrophilic. The surface of titanium dioxide is highly polar, and at high dioxide concentrations, the trend of the contact angle value towards the hydrophobicity can change.

**Figure 1:**
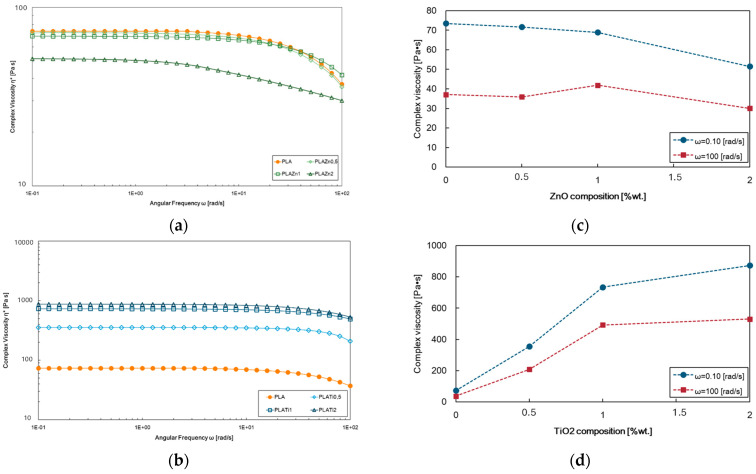
Rheological analysis, i.e., complex viscosity trends of (**a**) PLA/ZnO and (**b**) PLA/TiO_2_ at different contents, i.e., 0.5, 1, and 2%wt; (**c**,**d**) complex viscosity values at 0.1 and 100 rad/s.

Rheological characterization of the system provides information about processability and allows a better understanding of how the addition of fillers influences the structure–property relationship. Figure 1 displays the complex viscosity of neat PLA and PLA-based investigated samples. The pure polymer is mainly a linear long-chain structure, so it exhibits Newtonian behaviour in a wide range of frequencies, and the shear-thinning behaviour is observed for angular frequencies greater than 10 rad/s. The incorporation of ZnO at 0.5% and 1% does not cause significant variations, while the system PLA/ZnO at 2%wt shows a higher reduction in the complex viscosity values and more evident no-Newtonian behaviour (Figure 1a). These findings can be related to the decrease in the average molecular weight associated with the degradation of the PLA matrix during preparation due to significant ZnO presence [37]. Moreover, a dependence of the viscosity on the angular frequency is recorded (Figure 1b).

PLA filled with TiO_2_ differently exhibits a progressive rise in viscosity values concerning those of the neat matrix as the filler concentration increases (Figure 1c), and at lower dioxide contents, the angular frequency dependency is almost negligible, as shown in Figure 1d. The filled systems present an upward shift of the PLA’s complex viscosity curve and the onset of shear-thinning behaviour slightly moving to higher angular frequency values, suggesting that the matrix determines the rheological behaviour; the polymer–polymer interaction predominantly concerns poor polymer–filler interactions, also according to the literature [38,39]. The enhanced melt strength can further infer that the dioxide loading does not promote thermal degradation phenomena occurring in PLA processing.

**Figure 2:**
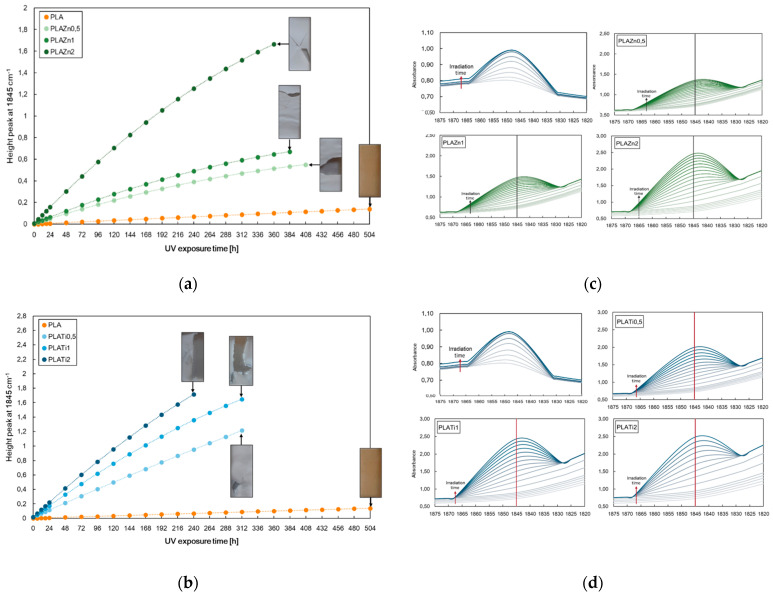
Height of the peak at 1845 cm^−1^ for neat PLA and (**a**) PLA/ZnO and (**b**) PLA/TiO_2_ at different contents, i.e., 0.5, 1, and 2%wt., and (**c**,**d**) show the selected zone of FTIR spectra of all investigated samples, used to elaborate the data in (**a**,**b**).

In Figure 2, the height of the peak at 1845 cm^−1^ for neat PLA and (a) PLA/ZnO and (c) PLA/TiO_2_ at different contents, i.e., 0.5, 1, and 2%wt, and the selected zone of FTIR spectra of all investigated samples are reported. 

According to the literature, the photo-oxidation of PLA is a complex phenomenon that occurs in forming anhydride functions that are detectable as shoulder-to-peak arising at 1845 cm^−1^. Further, the photo-oxidation behaviour of PLA-based nanocomposites can also be profitably monitored considering the changes in the same peak at 1845 cm^−1^ [40,41]. Considering these notions, the changes in the peak heights at 1845 cm^−1^ are followed for neat PLA and PLA containing different amounts of metal oxides; see Figure 2.

The neat PLA demonstrates excellent durability compared to the samples containing metal oxides. Interestingly, the investigated polymer–metal oxide nanocomposite has a higher degradation rate than that of the pristine matrix related to a superior photodegradation activity under UV light of the filled systems. The pro-degradant effect of both oxides on the polymer matrix increases as increases the filler amounts, and the degradation progress is faster for PLA/TiO_2_ than PLA/ZnO, also considering that thermal decomposition could occur during the preparation of PLA/ZnO systems. Photodegradability is affected by the diverse nature of the fillers, which absorb the energy and different dispersion and agglomeration of the fillers differently in the PLA-based system.

Adding to PLA, the 0.5 wt% of ZnO and TiO_2_ could be considered a feasible solution because the resultant material exhibits optimal physical properties, workability, and photodegradability rate for developing commercial products.

### 3.2. Effect of PA11 Adding on PLA Properties

Polymer blending is a practical and inexpensive approach in which two polymers are combined to achieve superior properties of materials. As reported in the literature, the composing polymers in PLA/PA11 blends exist in separate phases because of immiscibility, so the blend has been intensively studied with the purpose of improving the compatibility of PLA and PA11 [42,43,44,45,46]. Here, we investigate the effect of the incorporation by melt mixing of different amounts of PA11 in PLA polymer.

Table 2 reports the mechanical properties and water contact angle (WCA) measurements of neat PLA and PA11, and their blends in the analyzed composition range are as follows: PLA/PA11 = 85/15, 70/30, 50/50%wt/wt. The higher elastic modulus of PA11 with respect to PLA is attributed to intermolecular hydrogen bonding in amide groups present in both crystalline and amorphous phases [44].

The introduction of PA11 in PLA leads to deterioration in the elastic modulus and tensile strength values, and the blends exhibit a non-uniform worsening in mechanical performance with increasing PA11 content in the blends. The EB values of PLA decrease after the PA11 addition but become higher only for the 50/50%wt/wt blend. Moreover, PA11 causes a slight decrease in the surface hydrophilicity of PLA-based blends. The results suggest incompatibility and interfaces unable to successfully transfer the stresses between the polymers or tensions locally concentrated at defects such as voids. As the dispersion of the PA11 in the PLA matrix could influence stress concentrations, the morphological analysis and a statistical evaluation of the mean diameter of PA11 particles dispersed in the PLA matrix are later presented.

**Figure 3:**
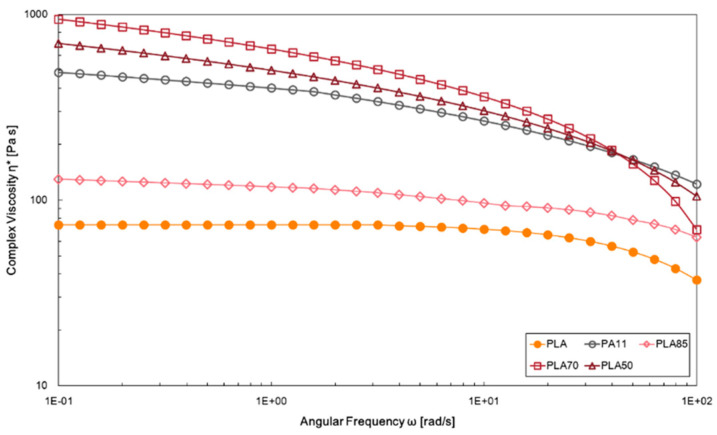
Complex viscosity of neat PLA and PLA/PA11 blends at 85/15, 70/30, and 50/50%wt/wt.

The complex viscosities values as a function of angular frequency for neat polymers and investigated blends are reported in Figure 3. The viscosity curve significantly differs between the neat PLA and the neat PA11; as discussed in the previous paragraph, PLA shows Newtonian behaviour in a wild range of frequency. Conversely, PA11 displays higher values of complex viscosity related to intermolecular interactions and shear-thinning behaviour across the whole investigated angular frequency range. At lower PA11 amounts in the blends, PLA seems to govern the rheological behaviour, while at higher PA11 amounts, rheological properties of the blend follow those of the dispersed phase, although, at higher frequencies, a more pronounced shear-thinning tendency is disclosed. The higher values of the complex viscosity in the low angular frequency range were recorded for the PLA/PA11 (70/30 wt/wt%) blend where the PLA chain mobility decreased; for this blend, a more severe viscosity drop is also observed in the high-frequency range.

**Figure 4:**
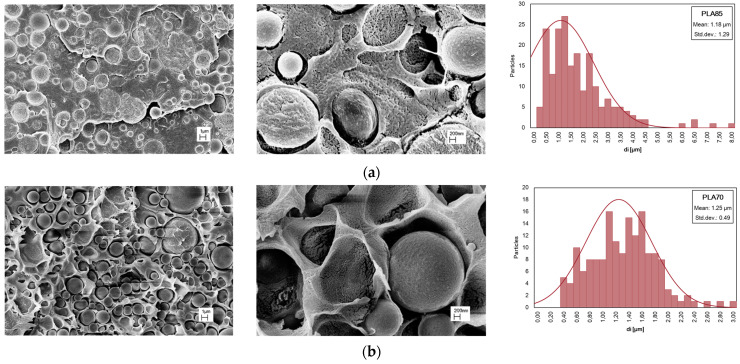

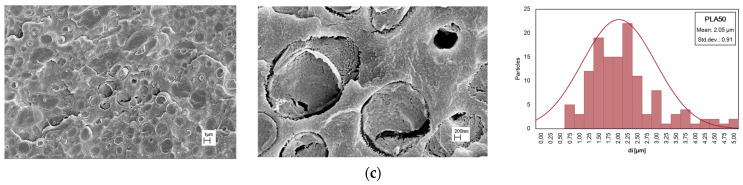
SEM observations at different magnifications and distribution of PA11 particle dimensions of PLA/PA11 blends at (**a**) 85/15, (**b**) 70/30, and (**c**) 50/50%wt/wt.

SEM micrographs of PLA/PA11 blends at various magnifications, different compositions, and distribution of PA11 particle dimensions are presented in Figure 4. Drop-matrix morphologies are obtained: the continuous phase is PLA, and the roughly spherical dispersed phase represents PA11. Even supposing PLA/PA11 miscible theoretical solubility, mainly related to the respective low and high polarity, the presence of voids at the PLA/PA11 interface can be noted, so the interface region could be the weakest point in the blend in which failure leading to fracture occurs. Also, according to the literature, the morphology of PLA/PA11, without adding nanoparticles and/or compatible agents, appears poor and the two biopolymer phases are distinguished. 

A statistical distribution of the dispersed phase size is reported in Figure 4, while in Table 3, the average diameter (*d_n_*) of PA11 particles in PLA and the ratio (*D*) between *d_n_* and *d_v_* in PLA/PA11 blends is published. As is known, the breakup and coalescence of dispersed droplets take place during polymer blend processing, and at high amounts of PA11, i.e., in the PLA/PA11 (50/50 wt/wt%) blend, the droplet coalescence phenomena dominate in the PLA matrix and the droplet shape changes from circular to elliptical. In this last event, the extension of the interface region decreases with respect to the blend containing a lower % of PA11 to minimize the free energy of the system, and the mechanical behaviour is mainly governed by the PLA matrix. Incrementing the PA11 content, the fractured surface shows a larger number of holes, suggesting that the adhesion between PLA and PA11 is very poor, and for this reason, PLA/PA11 = 50/50%wt/wt appears more fragile in comparison to other blends.

The photodegradation process of PLA and their considered blend was analyzed by monitoring the progress in the exposure time of 1845 cm^−1^ and 1725 cm^−1^ peaks, linked to the degradation pathway of PLA and PA11, respectively. According to the literature, the PA11 photo-oxidation process can be profitably monitored following the changes in heights of the peak at 1725 cm^−1^ as a function of UV irradiation time. As is known, this peak is assigned to the formation of new oxygen-containing species (e.g., carbonyl and carboxyl functionalities) [47].

**Figure 5:**
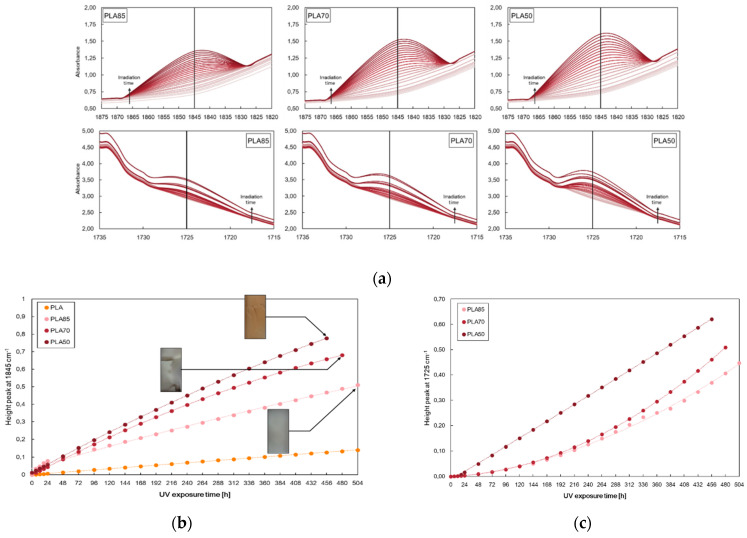
(**a**) Selected zone in FTIR spectra, (**b**) height of peak at 1845 cm^−1^, and (**c**) height of peak at 1725 cm^−1^ of neat PLA and of PLA/PA11 blends at 85/15, 70/30, and 50/50%wt/wt.

PLA, as discussed before, is a durable polymer during UV irradiation and degrades at a higher exposure time than PA11. Both photodegrade in the presence of oxygen by αH-abstraction and Norrish I and II photoreactions [44]. At low concentrations of PA11, although the photodegradation rate increases, the specimen endures for almost four weeks of analysis. Conversely, premature failure of the bio-blends containing higher PA11 concentrations is documented, and the photo resistance decreases as the PA11 amounts increase, as shown in Figure 5.

Therefore, it seems that the PLA/PA11 blends at 70/30%wt/wt could be subject to further investigation to obtain materials with designed properties at low cost.

### 3.3. Effect of ZnO and TiO_2_ Adding on PLA/PA11 (70/30%wt/wt) Properties

Nanoparticles added in an immiscible polymeric blend could enhance the rheological and mechanical properties of the blends, improve blend morphology, and stabilize the dispersed phase against coalescence [43,44,45]. Therefore, based on the above-discussed results and our previous experience, there are prepared biopolymer blends at PLA/PA11 = 70/30 wt/wt% containing ZnO and TiO_2_ at 0.5%wt.

Table 4 reports the mechanical properties of the PLA/PA11 blend and PLA/PA11-based blends. The PLA/PA11 blend with 0.5%wt of ZnO shows an improvement in the elastic modulus compared with the blend without oxide, no change in the tensile strength value, and a decrease in the elongation at break value; the water contact angle is 64.7° for the PLA/PA11 (70/30%wt/wt) blend and decreases to 53.5° for PLA/PA11 (70/30) + ZnO at 0.5%wt. Conversely, the presence of TiO_2_ in the blend did not positively influence the mechanical properties, although the elastic modulus value is higher compared to that of the neat blend, and a higher value for WCA is recorded. The results suggest the PA11 phase prevents the pro-degradant effect of the ZnO on the PLA phase, and we can also suppose that ZnO and TiO_2_ are more likely to be found at the interface of the dispersed phase.

**Figure 6:**
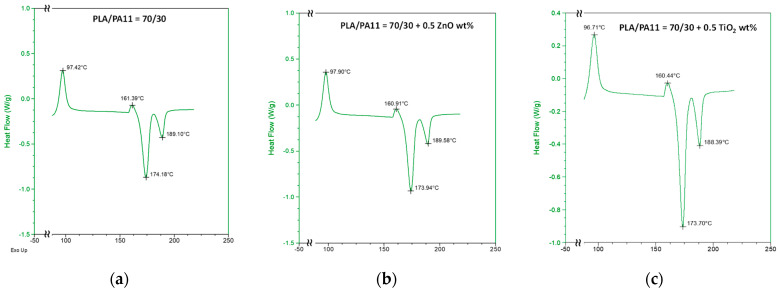
DSC trace of (**a**) PLA/PA11 (70/30%wt/wt) blend and PLA/PA11 blends containing (**b**) ZnO and (**c**) TiO_2_ at 0.5%wt.

Thermograms acquired by calorimetric analysis are reported in Figure 6. Cold PLA crystallization temperatures in the neat blend are about 97.4 and 161.4 °C; after ZnO was added at 0.5%wt to the blend, no significant changes concerning these temperatures and melting temperatures were observed. A supposed enhanced PLA chain mobility for TiO_2_ presence could instead cause slightly decreased cold crystallization and melting temperatures and a higher crystallinity degree.

**Figure 7:**
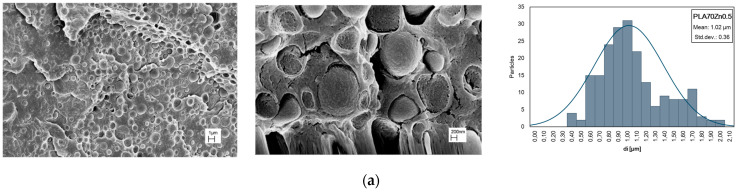

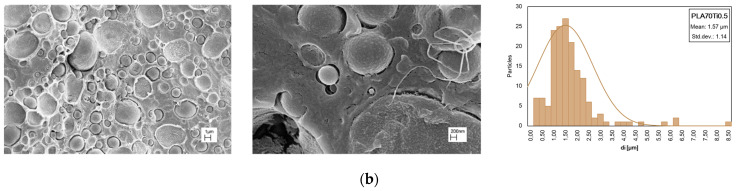
SEM observations at different magnifications and distribution of PA11 particle dimensions of PLA/PA11 blends containing (**a**) ZnO and (**b**) TiO_2_ at 0.5%wt.

It is crucial to analyze the effect of the nano-oxides’ incorporation on the morphology of the bio blend because of the important effect of the morphology of the blend on the final properties; furthermore, nano-oxides could reveal a networking ability and promote co-continuity in droplet–matrix morphologies. As expected, after the oxide additions at 0.5% to the blends, the morphology is still of the droplet–matrix type (Figure 7). Moreover, SEM micrographs point out that no oxide particles were found in the continuous phase. The presence of ZnO in the blend led to a PA11 droplet diameter reduction, indicating coalescence suppression and increased compatibility between polymers (see Table 3 and Figure 7a). We note that PA11-dispersed phase diameter seems to increase with respect to the neat blend after TiO_2_ introduction, and the narrowing of the size distribution is observed, as reported in Figure 7b.

**Figure 8:**
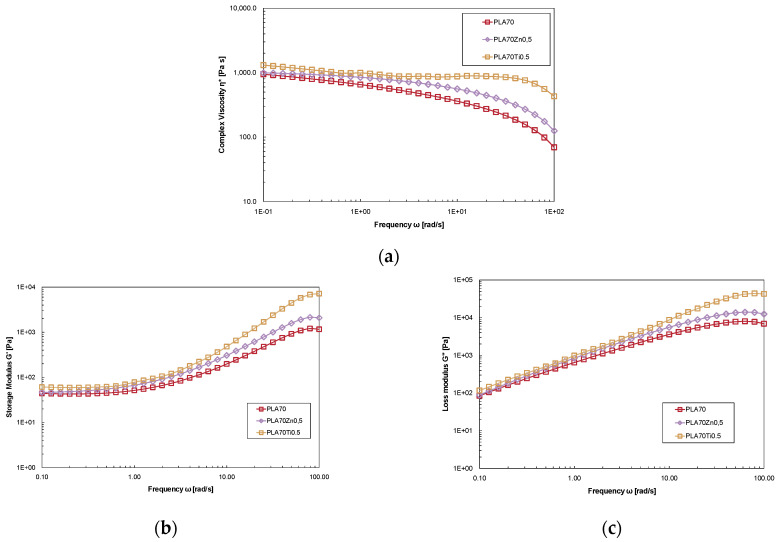
(**a**) Complex viscosity, (**b**) the storage modulus, G’, and (**c**) the loss modulus, G” of neat PLA/PA11 (70/30%wt/wt) blend and PLA/PA11 blends containing ZnO and TiO_2_ at 0.5%wt.

Figure 8 illustrates the complex viscosity η*, the storage modulus G’, and the loss modulus G” versus the angular frequency of the neat PLA/PA11 (70/30) blend and PLA/PA11 blends containing ZnO and TiO_2_ at 0.5%wt. The viscosity values of the PLA/PA11 (70/30) + ZnO at 0.5 wt% and of the PLA/PA11 (70/30) + TiO_2_ at 0.5%wt exceed those of the neat blend. In contrast to what was previously observed, the presence of ZnO does not cause lower complex viscosity of PLA because the PA11 presence prevents thermo-oxidative degradation during preparation. The blend containing TiO_2_ shows a similar viscosity curve to that of neat PLA. The Newtonian plateau of PLA/PA11 (70/30%wt/wt) + TiO_2_ at 0.5%wt is observed in a wider frequency range compared to the neat blend; moreover, at a low frequency, the loaded blend exhibits weak yield behaviour compared to the neat blend as a result of weak intermolecular force.

The rheological response of the storage modulus against the angular frequency of the investigated samples is illustrated in Figure 8b. The presence of both metal oxides in the blend enhances the modulus of the melts, indicating a reinforcing effect. The storage modulus for all samples is independent of the frequency in the low-frequency region, showing solid-like behaviour attributed to polymer interactions in the polymer matrix. Results obtained from the rheological analysis suggest that the rheological behaviour is mainly governed by the PLA matrix. Moreover, the enhancement of the rheological properties seems to confirm the affinity of both oxides towards the PA11 phase and the shielding effect of polyamide on PLA degradation against the pro-degradant action of metal oxide.

The effect of the investigated metal oxides upon UV irradiation depends on their distribution in the polymer matrix; in particular, they can act as pro-degradants, leading to a reduction in polymer molecular weight or can act as protecting agents, shielding UV irradiation [48,49]. Morphological, rheological, and mechanical analysis suggest that both oxides are tendentially localized in the PA11 phase or at the interface between PLA and PA11, thus avoiding the pro-degradation effect of ZnO on the PLA matrix. In any case, we must take into account that higher filler loadings could exceed a segregation limit in PA11 drops, causing their exposure towards the PLA phase. The PLA/PA11 blends containing ZnO and TiO_2_ at 0.5%wt. show very interesting photo-oxidation behaviour and were monitored again following the changes in both peaks at 1845 cm^−1^ and 1725 cm^−1^, related to the degradation pathways of PLA and PA11, respectively. By monitoring the height peak at 1845 cm^−1^, we can assert that the photodegradation rate of PLA, in terms of accumulation of anhydride functionalities, decreases in the presence of the blend of both ZnO and TiO_2_ nanoparticles. 

**Figure 9:**
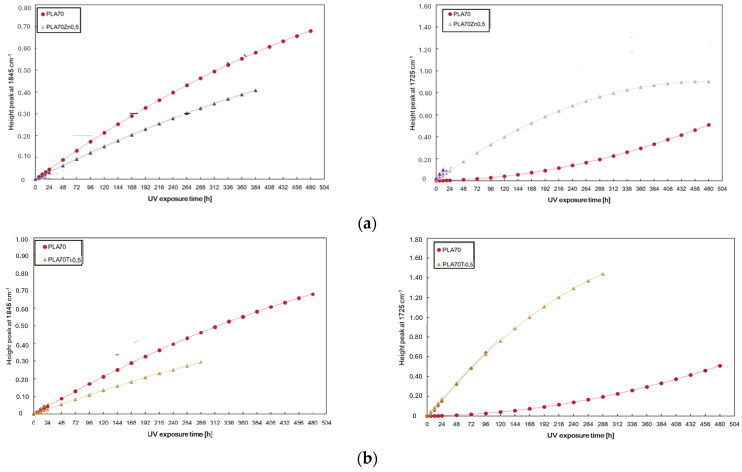
Photo-oxidation behaviour of neat PLA/PA11 (70/30) blend and PLA/PA11 blends containing (**a**) ZnO and (**b**) TiO_2_ at 0.5%wt.

On the contrary, the trends of the height of the peak at 1725 cm^−1^ show opposite trends, resulting in the accelerated photodegradation of PA11 in terms of the accumulation of oxygen-containing groups. The latter confirms that both ZnO and TiO_2_ are located preferentially in the more polar polyamide phase, causing its accelerated photodegradation. It is worth noting that both oxides cause a short lifetime of the obtained bio-blends. PLA/PA11/ZnO and PLA/PA11/TiO_2_ become brittle only after 384 h and 288 h of UV irradiation for ZnO and TiO_2_, respectively, in comparison to 480 h requested for neat PLA, and for this reason, the FTIR analyses were interrupted, as shown in Figure 9.

## 4. Conclusions

The properties of biopolymer and biopolymer blend-based nanocomposites are predominantly influenced by the individual nature of the constituents and the interface between them, as well as by the concentration, size, and shape of the particles and the morphology of the resultant system.

The presence of both metal oxides, namely ZnO and TiO_2_ at 0.5, 1, and 2%wt, in PLA leads to decreases in elastic modulus and tensile strength values, and this decrease is more pronounced for the samples containing ZnO, rather than the samples containing TiO_2_. This could be understood considering that the metal oxides also cause the degradation of PLA during melt processing. Both ZnO and TiO_2_ slightly increase the hydrophobicity of PLA, and the latter suggests that PLA hydrolysis could be slowed down. Further, both ZnO and TiO_2_ increase the formation of anhydride groups, which causes a significant reduction in PLA photo-oxidation resistance. Therefore, this work demonstrated that the incorporation of metal oxides into PLA-based matrices, although not desirable for the mechanical properties, improves the degradation efficiency of the nanocomposites, leading to a lower residence time of the resulting waste in the environment. The obtained results suggest that the optimal concentration for both metal oxides is 0.5%wt, and for this reason, this concentration is chosen to be introduced in the biopolymer blends.

Adding PA11 to the PLA matrix leads to the formation of brittle blends (lower values of elastic modulus, tensile strength, and elongation at break in comparison to that of neat PLA and PA11), and the best blend, PLA/PA11 = 70/30%wt/wt, shows best compromise between properties and resistance. As demonstrated, the PLA/PA11 blends, without metal oxides, show predominantly globular morphology that can be modified and refined by the presence of metal oxides up to ellipsoid morphology due to the preferential location of the particle in the more polar polyamide phase.

Regarding the photo-oxidation resistance of blends, interestingly, the PLA/PA11 blends containing ZnO and TiO_2_ show the slowed-down accumulation of anhydride species from the oxidation of PLA and accelerated accumulation of oxygen-containing groups from the degradation of PA11. The latter could be considered indirect evidence of the preferential location of the metal oxide particles in the more polar polyamide phase. 

The presence of both ZnO and TiO_2_ leads to a slight increase in elastic modulus and tensile strength values of the PLA/PA11 blend due to their reinforcement action. It could be concluded that the presence of both metal oxides has beneficial effects, although less pronounced, on the solid-state properties of the PLA/PA11 blend, helping to formulate biopolymer-based materials with modulated and tuned properties and performance. 

These findings aim to expand the use of PLA-based blends and composites, suggesting that further investigations, such as a compatibilizer presence or functionalization of the metal oxide particles, are required to improve and optimize the system morphology and properties.

## Figures and Tables

**Table 1:** Mechanical properties: Young’s modulus (E), tensile strength (TS), elongation at break (EB), water contact angle (WCA) measurements, and crystallinity degree (Δ) of neat PLA, PLA/ZnO, and PLA/TiO_2_ at different contents, i.e., 0.5, 1, and 2%wt.

Samples	E, MPa	TS, MPa	EB, %	WCA, deg	Δ, %
PLA	980 ± 65	34.9 ± 2.5	6.2 ± 0.5	60.10 ± 1.0	9.5
PLA + ZnO at 0.5%wt	542 ± 52	33.3 ± 3.1	3.5 ± 0.4	65.15 ± 1.2	6.1
PLA + ZnO at 1%wt	424 ± 45	29.8 ± 3.0	2.9 ± 0.3	70.19 ± 1.3	5.8
PLA + ZnO at 2%wt	199 ± 22	22.9 ± 3.1	3.6 ± 0.3	78.61 ± 1.2	4.3
PLA + TiO_2_ at 0.5%wt	741 ± 68	35.9 ± 3.4	7.9 ± 0.5	62.64 ± 1.1	6.7
PLA + TiO_2_ at 1%wt	750 ± 69	29.1 ± 3.2	8.7 ± 0.5	65.18 ± 1.2	5.2
PLA + TiO_2_ at 2%wt	744 ± 65	22.5 ± 2.7	9.3 ± 0.6	53.90 ± 1.0	4.8

**Table 2:** Mechanical properties, i.e., Young’s modulus (E), tensile strength (TS), elongation at break (EB) and water contact angle (WCA) measurements of neat PLA, neat PA11, and their blends (i.e., PLA/PA11 = 85/15, 70/30, and 50/50%wt/wt.)

Samples	E, MPa	TS, MPa	EB, %	WCA, deg
PLA	980 ± 65	34.9 ± 2.5	6.2 ± 0.5	60.10 ± 1.0
PA11	1250 ± 110	19.7 ± 1.5	22.8 ± 2.5	85.3 ± 2.3
PLA/PA11 (85/15%wt/wt)	632 ± 65	12.7 ± 1.2	2.1 ± 0.3	62.4 ± 1.2
PLA/PA11 (70/30%wt/wt)	579 ± 54	14.8 ± 1.5	5.0 ± 0.7	64.7 ± 1.0
PLA/PA11 (50/50%wt/wt)	828 ± 77	21.5 ± 2.1	8.2 ± 0.8	64.7 ± 1.1

**Table 3:** Average diameter (*d_n_*) of PA11 particles in PLA and ratio (*D*) between dn and dv in PLA/PA11 blends and blends containing metal oxides, i.e., ZnO and TiO_2_ at 0.5%wt.

Samples	Σn_i_	$d_{n}[\mu m]$	D
(a)			
PLA/PA11 (85/15%wt/wt)	201	1.28	3.46
PLA/PA11 (70/30%wt/wt)	166	1.35	1.41
PLA/PA11 (50/50%wt/wt)	129	2.05	1.65
(b)			
PLA/PA11 (70/30) + ZnO at 0.5%wt	204	1.02	1.36
PLA/PA11 (70/30) + TiO_2_ at 0.5%wt	132	1.57	3.50

**Table 4:** Mechanical properties, i.e., Young’s modulus (E), tensile strength (TS) and elongation at break (EB) and water contact angle (WCA) measurements of PLA/PA11 (70/30%wt/wt) blend and PLA/PA11 blends containing ZnO and TiO_2_ at 0.5 wt%.

Samples	E, MPa	TS, MPa	EB, %	WCA, deg
PLA/PA11 (70/30%wt/wt)	579 ± 54	14.8 ± 1.5	5.0 ± 0.7	64.7 ± 1.0
PLA/PA11 + ZnO at 0.5%wt	662 ± 63	14.8 ± 1.4	4.4 ± 0.4	53.5 ± 1.2
PLA/PA11+ TiO_2_ at 0.5%wt	589 ± 51	9.2 ± 0.9	3.9 ± 0.3	66.7 ± 1.4

## Data Availability

Data are contained within the article.
